# Changes in Perceived Exertion, Well-Being, and Recovery During Specific Judo Training: Impact of Training Period and Exercise Modality

**DOI:** 10.3389/fphys.2020.00931

**Published:** 2020-08-14

**Authors:** Ibrahim Ouergui, Luca Paolo Ardigò, Okba Selmi, Danielle Evé Levitt, Hamdi Chtourou, Anissa Bouassida, Ezdine Bouhlel, Emerson Franchini

**Affiliations:** ^1^High Institute of Sports and Physical Education of Kef, University of Jendouba, El Kef, Tunisia; ^2^Department of Neurosciences, Biomedicine and Movement Sciences, School of Exercise and Sport Science, University of Verona, Verona, Italy; ^3^Applied Physiology Laboratory, University of North Texas, Denton, TX, United States; ^4^Department of Physiology, School of Medicine, Louisiana State University Health Sciences Center New Orleans, New Orleans, LA, United States; ^5^Institut Supérieur du Sport et de l’Education Physique de Sfax, Université de Sfax, Sfax, Tunisia; ^6^Activité Physique, Sport et Santé, UR18JS01, Observatoire National du Sport, Tunis, Tunisia; ^7^Laboratory of Cardio-Circulatory, Respiratory, Metabolic and Hormonal Adaptations to Muscular Exercise, Faculty of Medicine Ibn El Jazzar, University of Sousse, Sousse, Tunisia; ^8^Martial Arts and Combat Sports Research Group, School of Physical Education and Sport, University of São Paulo, São Paulo, Brazil

**Keywords:** overreaching, overtraining, judo, training load, recovery, taper

## Abstract

The present study investigated the effect of intense and tapering training periods using different exercise modalities (i.e., Randori – grip dispute practice without throwing technique, Uchi-komi – technique repetition training, and sprinting) on rating of perceived exertion (RPE), well-being indices, recovery state, and physical enjoyment in judo athletes. Sixty-one adolescent male and female judo athletes (age: 15 ± 1 years) were randomly assigned to one of three experimental or one control groups. Experimental groups (Randori, Uchi-komi, and running) trained four times per week for 4 weeks of intense training (in addition to their usual technical-tactical judo training; control group underwent only such a training) followed by 12 days of tapering. RPE, well-being indices [i.e., sleep, stress, fatigue, and delayed onset muscle soreness (DOMS)], total quality of recovery (TQR), and physical enjoyment were measured every session. RPE, sleep, stress, fatigue, DOMS, Hooper index (HI; sum of wellbeing indices), and TQR were lower in the tapering compared with the intensified training period (*P* < 0.001). Moreover, the running group showed better values for sleep (*P* < 0.001), stress (*P* < 0.001), fatigue (*P* = 0.006), DOMS (*P* < 0.001), and HI (*P* < 0.001) in comparison with the other training groups, indicating a more negative state of wellbeing. The Randori and Uchi-komi groups showed higher values for TQR and physical enjoyment (both *P* < 0.001) than the running group, whereas RPE was lower in the control compared with all training groups (*P* < 0.001). Coaches should use more specific training modalities (i.e., Randori and Uchi-komi) during intensified training and should monitor well-being indices, RPE, and TQR during training periods. Moreover, for all variables, 12 days tapering period are beneficial for improving wellbeing and recovery after 4 weeks of intense training.

## Introduction

Judo is a dynamic grappling combat sport characterized by high-intensity intermittent actions, requiring complex skills and tactical excellence for success (Franchini et al., 2011). It has been reported that judo athletes need to predominantly develop aerobic metabolism to supply sufficient energy throughout the 4-min judo match, whereas the high-intensity scoring and defensive actions are mainly supported by anaerobic high-energy phosphate and glycolytic energy systems (Julio et al., 2017). Each energy system responds best to different training stimuli, and each supports different physical components of judo matches. Therefore, many studies have been conducted to examine the effect of different training modes to enhance physiological aspects that are directly related to success in judo competition (Papacosta et al., 2013; Franchini et al., 2016a, b; Ouergui et al., 2019).

Among these modalities, high-intensity intermittent training (HIIT) has emerged as a training *regimen* commonly used for judoka athletes. This training modality has demonstrated efficacy in developing both aerobic and anaerobic performance (Franchini et al., 2016a, b). Although effective, HIIT imposes heavy training loads on athletes and therefore must be well controlled because an increase in training loads coupled with insufficient recovery between training sessions may cause non-functional overreaching and injury (Magnani Branco et al., 2017). Furthermore, it was reported that intensified training (i.e., increased training loads) is related to psychometric *status* in athletes, which can be influenced by workload, fatigue accumulation, imbalance between training and recovery, and training modality (Selmi et al., 2018b).

Intense training generally induces significant neuromuscular, physiological, and hormonal responses that lead to changes in well-being and recovery state (Hooper et al., 1995). Furthermore, tapering, or a brief period of reduced physical demands usually performed after an intense training period, is used across many sports and allows for manifestation of mental and physical performance improvements after being negatively affected by a previous intense training period (Papacosta et al., 2013). For that reason, markers of well-being and recovery state associated with intense training and tapering periods have received much attention in recent years (Papacosta et al., 2013; Selmi et al., 2019). However, well-being and recovery have not been examined during intense training and tapering periods in judo.

Self-report questionnaires are widely used in sport to determine perceived intensity, well-being, and recovery state and to monitor physiological and psychometric *status* of athletes (Selmi et al., 2019). To monitor well-being, Hooper and Mackinnon (1995) developed a set of scales to assess sleep quality, fatigue level, stress, and delayed onset muscular soreness (DOMS). These scales are sensitive to higher training loads and intensified training (i.e., high stress, poor sleep quality, fatigue, and higher DOMS; Moalla et al., 2016). Moreover, Kenttä and Hassmén (1998) developed the total quality recovery (TQR) scale to assess recovery state between training sessions. It was shown that TQR score can be influenced by increased training loads (Freitas et al., 2014), recovery state and muscular fatigue (Osiecki et al., 2015), and that athletic performance may be affected by the quality of recovery (Selmi et al., 2019).

Furthermore, the level of perceived physical enjoyment during training influences training adherence. When intensified training is perceived as enjoyable, it may produce positive mood states, improved *vigor*, and increased motivation in athletes (Selmi et al., 2018b). Recent studies reported that exercise mode affects perceived enjoyment, but enjoyment is not necessarily associated with physiological demands of training session, subjective training intensity, or recovery quality (Thum et al., 2017; Selmi et al., 2019). Training monitoring studies have indicated that young judo athletes (cadets and juniors) cope well with increased effort when a periodized training program is used (Agostinho et al., 2015, 2017) but studies verifying the effects of intensified training and a subsequent tapering period on rating of perceived exertion (RPE), well-being indices and recovery state, and physical enjoyment are lacking. Such studies may help coaches and strength and conditioning professionals to better understand the impact of intensified training in these athletes, allowing for improved training monitoring and prescription.

To the authors’ knowledge, no previously published study has investigated the changes in well-being indices and recovery state during intense training and tapering periods in judo athletes. These athletes’ recovery *status* is very important within their health and performance management. Coaches need to optimize recovery achievement by carefully controlling training load (Bromley et al., 2018). Thus, the aim of this study was to investigate the effect of intense and tapering training periods using different exercise modalities on RPE, well-being indices, recovery state, and physical enjoyment in judo athletes.

## Materials and Methods

### Participants

Sixty-one judo athletes (male, *n* = 35; and female, *n* = 26) volunteered to participate in the study. Using an effect size of 0.3 based on the study of Franchini et al. (2016b) and the software G^∗^Power with α and power fixed at 0.05 and 0.95, respectively, the resulting *a priori* sample size was 40. The athletes were randomly assigned to either a Randori [viz. grip dispute practice without throwing technique, RG, males *n* = 8 and females *n* = 6, age 16 ± 1 years, height 171.0 ± 0.8 cm, and body mass 58.4 ± 7.4 kg (mean ± SD)], Uchi-komi (i.e., technique repetition training, UG, males *n* = 10 and females *n* = 6, age 15 ± 1 years, height 164.2 ± 0.8 cm, and body mass 58.9 ± 8.3 kg), running (RuG, male *n* = 9 and female *n* = 8, age 15 ± 1 years, height 162.5 ± 1.1 cm, and body mass 55.8 ± 3.2 kg), or a control group (CG, males *n* = 6 and females *n* = 6, age 15 ± 1 years, height 159.2 ± 0.5 cm, and body mass 56.8 ± 5.4 kg). Randomization was performed using a random assignments Excel spreadsheet (Excel 2007, Microsoft Office, Microsoft, Redmond, WA, United States). The control group performed regular judo training consisting of a technical-tactical judo training routine. Experimental groups were administered HIIT sessions, twice per week. Additionally, participants of all groups trained four sessions per week (1 h 30 min/session) and had more than 7 years of experience in judo practice, participating regularly in regional and national competitions for at least 2 years prior to the enrolment in the study. Athletes reported no medical restrictions during the experimental period. The study was conducted according to the Declaration of Helsinki for human experimentation (World Medical Association, 2013) and the protocol was fully approved by the local research ethics committee before the start of the study. All the athletes, their parents, and coaches gave written informed consent after a detailed explanation of the aims and risks involved in the investigation.

### Methodology

**FIGURE 1:**
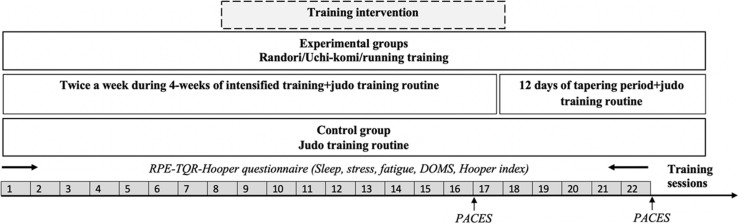
Schematic representation of study design. RPE, rating of perceived exertion; TQR, total quality of recovery; DOMS, delayed onset muscle soreness; PACES, physical activity enjoyment scale.

One week before the beginning of the experimental period, athletes were familiarized with the testing procedures including all scales and questionnaires. Athletes were randomly assigned either to a control group (CG) or to one of three experimental groups: Randori (RG), Uchi-komi (UG), and running (RuG; Figure 1). In order to control main growth variables, height and body mass were compared among different groups. Athletes in experimental groups completed HIIT training in their assigned training modality twice a week during a 4-week intense training period in addition to their usual technical-tactical judo training. The control group executed only the usual judo training without additional HIIT. Following the intense training period, athletes completed 12 days of tapering where HIIT volume was reduced. Before each training session, perceived recovery was assessed by the TQR scale and well-being indices scores (i.e., stress, sleep quality, fatigue, and DOMS). Then, athletes completed a 15-min standardized warm-up consisting of jogging, dynamic stretching, and specific skills (e.g., technique entrance). Following the warm-up, athletes carried out their assigned training for that day. After each training session, athletes indicated their RPE for that session. Moreover, physical enjoyment was assessed at the ends of the intensified and tapering training periods using the physical activity enjoyment scale (PACES). Training sessions and all measures were conducted at the same time of day and the study took place during the competitive period of the season.

### Measures

#### High-Intensity Intermittent Training Program

During the intense training period, each HIIT training session lasted 14 min 40 s, not including warm-up and cool-down phases. The workouts were composed of two blocks of 4 min 50 s of HIIT exercises (10 × 20-s efforts separated by 10-s rest breaks). Athletes recovered for 5 min between blocks (Franchini et al., 2016a, b). During the tapering period, HIIT volume was reduced to only one block. Athletes performed each session using their assigned training modality at an “all-out” intensity (Franchini et al., 2016b). For the Randori group, athletes were grouped by weight categories and asked to spar in a standing combat phase ensuring the Kumi-kata (i.e., grip dispute) with attempts to execute techniques without throwing. For the Uchi-komi group, athletes were asked to perform repetitive technical entrance without throwing, with a single partner from the same weight category. Uchi-komi refers to the repeated practice of executing the technique without throwing and with a single partner in stationary position. For the running group, athletes were asked to run back and forth as fast as possible between two lines separated by 6 m as used in a specific judo fitness test (Sterkowicz et al., 1999). Regular judo training was carried out after HIIT during both training periods.

#### Subjective Well-Being and Recovery State

Athletes completed the well-being questionnaire (Hooper and Mackinnon, 1995) and TQR scale 15 min before each training session to assess well-being and recovery state. The well-being questionnaire consists of four items (stress, sleep quality, fatigue, and DOMS) and sum of these four variables’ scores was used to calculate the Hooper index (HI). Higher scores indicate more stress, fatigue, and DOMS, worse sleep quality, and poorer overall recovery. TQR scale was used to assess athletes’ recovery state and was reflective of the response to the preceding training day (Kenttä and Hassmén, 1998). Higher TQR scores reflect better recovery.

#### Rating of Perceived Exertion

Rating of perceived exertion was recorded 15–30 min after each training session using the Borg CR-10 RPE scale (adapted by Foster et al., 2001).

#### Physical Activity Enjoyment Scale

The PACES questionnaire was used to assess physical enjoyment (Kendzierski and DeCarlo, 1991). This scale contains 18 items and the overall physical activity enjoyment score was generated by summing the individual item scores.

### Statistical Analysis

Data are presented as mean ± standard deviation. Statistical analyses were performed using SPSS 20.0 statistical software (SPSS Inc., Chicago, IL, United States). Normality of data sets was verified using the Kolmogorov–Smirnov test. Sphericity was tested using the Mauchly test. A two-way (group × time-point) analysis of variance (ANOVA) with repeated measures on time-point was used. A Bonferroni *post hoc* test was applied to detect the location of any main or interaction effects. Standardized effect size (Cohen’s *d*) analysis was used to interpret magnitude of differences between variables and classified according to Hopkins^1^. Moreover, upper and lower 95% confidence intervals of the difference (95% CI_*d*_s) were calculated for the corresponding variation. Significance level was set at *P* ≤ 0.05.

## Results

Regarding height, there was a significant difference among groups (*F*_3,55_ = 465.07 and *P* < 0.001), with Randori athletes taller than Uchi-komi ones (*P* = 0.0017). Uchi-komi athletes were taller than running and control ones (*P* < 0.001 for both comparisons). Likewise, running athletes were taller in comparison with control ones (*P* < 0.001). Differently, body mass was not different among groups (*F*_3,55_ = 0.8026 and *P* = 0.4978).

Rating of perceived exertion differed between groups (*F*_3,1254_ = 31.354 and *P* < 0.001, Table 1), with lower values for control compared with Randori (*P* < 0.001), Uchi-komi (*P* < 0.001), and running (*P* < 0.001)^2^. There was also a time-point effect (*F*_21,1254_ = 7.109 and *P* < 0.001), with higher values for 1st in comparison with 18th (*P* = 0.001), 19th (*P* = 0.002), 20th (*P* < 0.001), 21st (*P* < 0.001, respectively), and 22nd (*P* = 0.011) training sessions; higher values for 3rd in comparison with 18th training session (*P* = 0.006); higher values for 2nd (*P* = 0.0032), 8th and 9th (*P* = 0.01 and *P* = 0.023, respectively), and 15th and 16th (*P* = 0.01 and 0.006, respectively) in comparison with 20th training session. Moreover, there were higher values for 10th and 11th in comparison with 18th (*P* = 0.001), 19th (*P* = 0.005 and *P* < 0.001, respectively), 20th (*P* < 0.001 for both comparisons), 21st (*P* < 0.001 for both comparisons), and 22nd (*P* = 0.0027 and *P* < 0.001, respectively) training sessions; higher values for 12th and 14th in comparison with 18th (*P* = 0.002 and *P* = 0.001, respectively), 19th (*P* = 0.003 and *P* = 0.002, respectively), 20th (*P* < 0.001 for both comparisons), 21st (*P* < 0.001 for both comparisons), and 22nd (*P* < 0.001 and *P* = 0.013, respectively) training sessions.

**TABLE 1:** Total quality of recovery and perceived exertion measured during intensified training and tapering periods in Randori (RG), Uchi-komi (UG), running (RuG), and control (CG) groups (mean ± SD; RG *n* = 14; UG *n* = 16; RuG *n* = 17; and CG *n* = 12).

	Total quality of recovery (a.u.)				Rating of perceived exertion (a.u.)			
	RG	UG	RuG	CG	RG	UG	RG	CG
S1	14 ± 2	15 ± 2	15 ± 2	15 ± 2	5 ± 2^a^	4 ± 1^a^	5 ± 1^a^	3 ± 2^a^
S2	15 ± 2	15 ± 2	14 ± 2	15 ± 3	4 ± ^c^	4 ± 1^c^	4 ± 1^c^	3 ± 1^c^
S3	15 ± 2	15 ± 2	14 ± 2	15 ± 4	5 ± ^b^	4 ± 1^b^	5 ± 1	2 ± 1^b^
S4	14 ± 2	14 ± 2	15 ± 2	16 ± 2	4 ± 1	4 ± 1	4 ± 1	3 ± 1
S5	15 ± 3	14 ± 2	14 ± 2	15 ± 3	4 ± 1	4 ± 1	5 ± 2	3 ± 1
S6	15 ± 3	15 ± 2	15 ± 2	16 ± 3	4 ± 1	4 ± 1	5 ± 2	3 ± 1
S7	16 ± 2	16 ± 1	15 ± 2	15 ± 3	4 ± 1	3 ± 1	4 ± 1	3 ± 2
S8	16 ± 2*^$^*	16 ± 2*^$^*	16 ± 1*^$^*	16 ± 3*^$^*	4 ± 1	4 ± 1	4 ± 2	3 ± 1
S9	15 ± 2	15 ± 1	16 ± 1	15 ± 3	4 ± 1	4 ± 1	3 ± 1	4 ± 2
S10	14 ± 2^¶^	14 ± 3^¶^	14 ± 3^¶^	16 ± 3^¶^	4 ± 1^e^	4 ± 1^e^	5 ± 2^e^	3 ± 2^e^
S11	16 ± 2	15 ± 2	14 ± 3	15 ± 4	4 ± 1^e^	4 ± 1^e^	5 ± 2^e^	4 ± 1^e^
S12	16 ± 2	15 ± 2	14 ± 3	15 ± 4	4 ± 1^e^	4 ± 1^e^	5 ± 1^e^	4 ± 2^e^
S13	14 ± 3	14 ± 2	14 ± 2	16 ± 3	4 ± 1	4 ± 1	4 ± 1	3 ± 1
S14	14 ± 3	15 ± 2	14 ± 2	15 ± 3	4 ± 1	4 ± 1^e^	4 ± 21^e^	4 ± 2^e^
S15	15 ± 2	16 ± 2	15 ± 2	15 ± 2	4 ± 1	4 ± 1	4 ± 1	3 ± 1
S16	15 ± 2	16 ± 1	14 ± 2	15 ± 1	4 ± 1	4 ± 1	4 ± 1	3 ± 1
S17	15 ± 2	15 ± 2	14 ± 3	14 ± 1	4 ± 1	4 ± 1	4 ± 1	3 ± 1
S18	16 ± 1	16 ± 1	15 ± 2	15 ± 1	3 ± 1	3 ± 1	3 ± 1	3 ± 1
S19	16 ± 2	17 ± 1	15 ± 3	15 ± 2	3 ± 1	3 ± 1	3 ± 1	3 ± 1
S20	16 ± 2	16 ± 1	15 ± 2	16 ± 2	3 ± 1^d^	1 ± 1^d^	3 ± 1^d^	3 ± 1^d^
S21	16 ± 1	16 ± 1	16 ± 1	15 ± 3	3 ± 1	1 ± 1	3 ± 1	3 ± 1
S22	16 ± 1^§^	16 ± 1^§^	16 ± 2^§^	16 ± 2^§^	4 ± 1	4 ± 1	3 ± 1	3 ± 1
Overall	15 ± 2	15 ± 2	14 ± 2^∗^	15 ± 3	4 ± 1	4 ± 1	4 ± 1	3 ± 1^†^

*^∗^Different from RG, UG, and CG; ^§^Different from 1st, 3rd, 5th, 10th, 13th, and 17th training sessions; *^$^*Different from 10th session; ^¶^Different from 18th to 21st training sessions; ^†^Different from RG, UG, and RuG; ^*a*^Different from 18th, 19th, 20th, 21st, and 22nd training sessions; ^*b*^Different from 18th training session; ^*c*^Different from 8th to 9th training sessions; ^*d*^Different from 15th to 16th training sessions; ^*e*^Different from 18th, 19th, 20th, 21st, and 22nd training sessions; a.u., arbitrary unit; S, training session.*

Sleep quality was different between groups (*F*_3,1254_ = 6.164 and *P* < 0.001, Table 2). Higher values (i.e., poorer sleep quality) were observed for running than for Randori (*P* < 0.001) and control (*P* = 0.038). Moreover, there was a time-point effect (*F*_21,1254_ = 1.599 and *P* = 0.042) with higher values for 1st compared with 7th (*P* = 0.002) and 8th training sessions (*P* = 0.012).

**TABLE 2:** Well-being indices measured during intensified training and tapering periods in Randori (RU), Uchi-komi (UG), running (RuG), and control (CG) groups (mean ± SD; RG *n* = 14; UG *n* = 16; RuG *n* = 17; and CG *n* = 12).

	Sleep (a.u.)				Stress (a.u.)				Fatigue (a.u.)				DOMS (a.u.)				Hooper Index (a.u.)			
	RG	UG	RuG	CG	RG	UG	RuG	CG	RG	UG	RuG	CG	RG	UG	RuG	CG	RG	UG	RuG	CG
S1	2.9 ± 1.3^¶^	2.9 ± 1.3^¶^	3.1 ± 1.1^¶^	2.5 ± 1.7^¶^	2.8 ± 1.8*^$^*	2.9 ± 1.1*^$^*	3.6 ± 1.1*^$^*	3.1 ± 1.0*^$^*	3.4 ± 0.7^c^	3.1 ± 0.5^c^	3.6.±1.1^c^	3.2 ± 1.6^c^	3.3 ± 1.1	2.9 ± 1.1	3.6 ± 0.9	2.4 ± 1.4	12.4 ± 2.3^j^	11.9 ± 2.3^j^	14.2 ± 3.8^j^	11.2 ± 4.4^j^
S2	2.4 ± 0.8	29 ± 0.9	2.8 ± 1.0	2.1 ± 1.3	2.6 ± 1.3	2.6 ± 1.3	3.4 ± 1.3	3.2 ± 1.2	2.9 ± 0.7	2.7 ± 0.7	3.4 ± 1.0	2.7 ± 1.1	3.1 ± 0.9	2.9 ± 1.2	3.4 ± 1.4	2.7 ± 1.1	11.1 ± 2.0	11.1 ± 2.8	13.0 ± 3.5	10.7 ± 3.5
S3	2.4 ± 1.0	2.6 ± 1.3	2.7 ± 1.1	2.5 ± 1.5	2.1 ± 0.9	2.5 ± 1.1	2.7 ± 0.6	2.7 ± 1.4	2.4 ± 0.8	2.7 ± 1.1	2.9 ± 0.7	2.2 ± 1.1	3.4 ± 1.3	3.4 ± 1.4	3.2 ± 1.1	1.8 ± 1.0	10.4 ± 3.5	11.1 ± 3.3	11.5 ± 2.1	9.2 ± 3.9
S4	2.3 ± 0.6	2.6 ± 0.6	2.5 ± 1.0	1.9 ± 0.8	2.6 ± 0.6	2.4 ± 0.7	3.3 ± 0.7	1.8 ± 0.8	2.9 ± 0.9	3.0 ± 0.7	3.2 ± 1.2	2.6 ± 1.3	3.4 ± 1.4	3.4 ± 0.9	3.3 ± 1.4	2.5 ± 1.4	11.1 ± 2.5	11.4 ± 2.2	12.2 ± 4.7	8.8 ± 2.9
S5	2.6 ± 0.5	2.8 ± 1.0	2.6 ± 1.1	2.2 ± 1.0	2.1 ± 0.5	2.6 ± 0.7	3.2 ± 1.5	2.5 ± 1.1	3.4 ± 0.7	3.3 ± 0.6	2.9 ± 1.2	2.3 ± 1.0	3.2 ± 1.3^f^	3.2 ± 0.9^f^	3.0 ± 1.3^f^	3.1 ± 2.1^f^	11.4 ± 2.2	11.8 ± 2.1	11.8 ± 4.6	9.8 ± 3.9
S6	2.7 ± 1.4	2.9 ± 1.0	2.7 ± 1.0	2.4 ± 1.2	2.5 ± 0.8^a^	2.2 ± 0.6^a^	3.0 ± 1.3^a^	2.7 ± 0.9^a^	2.8 ± 0.9^d^	2.6 ± 0.7^d^	2.9 ± 0.8^d^	2.7 ± 1.0^d^	2.9 ± 1.2	2.8 ± 0.9	3.3 ± 1.0	2.7 ± 1.7	10.6 ± 3.4	10.4 ± 2.2	12.0 ± 3.4	10.5 ± 3.6
S7	1.9 ± 0.8	2.0 ± 0.9	1.8 ± 0.7	2.5 ± 1.1	1.7 ± 0.7^§^	1.6 ± 0.9^§^	2.8 ± 1.2^§^	2.8 ± 0.8^§^	2.4 ± 0.7	2.4 ± 0.6	2.0 ± 1.0	2.7 ± 1.0	3.1 ± 0.7	3.0 ± 0.6	2.8 ± 0.9	2.8 ± 1.4	9.2 ± 2.4^k^	9.0 ± 2.0^k^	9.7 ± 2.8^k^	10.9 ± 2.8^k^
S8	1.9 ± 0.8	2.2 ± 0.7	2.05 ± 0.62	2.4 ± 0.8	1.9 ± 0.9	1.6 ± 0.6	3.1 ± 1.6	2.7 ± 0.9	2.4 ± 0.6	2.8 ± 1.2	2.6 ± 1.2	2.7 ± 1.4	2.9 ± 0.7	3.1 ± 0.9	2.9 ± 1.1	2.9 ± 1.6	9.1 ± 1.8	9.8 ± 2.8	10.7 ± 3.3	10.7 ± 3.6
S9	2.2 ± 0.7	2.6 ± 0.9	2.6 ± 0.9	2.5 ± 1.2	2.1 ± 0.8	2.0 ± 0.7	2.2 ± 0.8	3.2 ± 1.1	3.1 ± 1.2	2.6 ± 1.0	2.6 ± 1.0	3.0 ± 1.6	3.7 ± 1.3	3.1 ± 1.2	2.8 ± 1.1	3.3 ± 1.5	11.2 ± 2.7	10.2 ± 1.9	10.2 ± 2.2	12.1 ± 3.5
S10	2.43 ± 0.58	2.63 ± 0.89	2.68 ± 1.34	2.6 ± 1.4	2.7 ± 0.8	2.7 ± 0.9	3.5 ± 1.5	3.2 ± 0.9	2.8 ± 1.1	3.2 ± 1.1	2.7 ± 1.2	3.2 ± 1.3	3.5 ± 1.2^g^	3.7 ± 1.2^g^	3.2 ± 1.2^g^	3.1 ± 1.6^g^	11.5 ± 3.1	12.2 ± 3.4	11.95 ± 4.0	12.0 ± 3.2
S11	2.0 ± 0.8	2.2 ± 1.0	2.6 ± 1.1	2.2 ± 1.1	2.0 ± 0.9	2.2 ± 0.9	3.4 ± 1.6	3.4 ± 1.4	2.6 ± 0.6	2.6 ± 0.6	3.0 ± 1.3	2.7 ± 1.2	3.5 ± 0.6	3.2 ± 0.7	3.6 ± 1.2	3.0 ± 1.6	10.2 ± 2.5	10.25 ± 2.65	12.37 ± 3.93	11.3 ± 4.2
S12	2.0 ± 0.9	2.5 ± 0.7	2.8 ± 0.9	2.5 ± 1.1	2.0 ± 09	2.4 ± 0.8	3.2 ± 1.3	2.7 ± 1.0	2.5 ± 0.9	2.9 ± 1.0	3.2 ± 0.9	2.3 ± 1.1	3.1 ± 1.0	3.4 ± 1.2	3.2 ± 0.8	2.7 ± 1.1	9.5 ± 3.0	11.1 ± 3.4	12.0 ± 3.7	10.0 ± 2.8
S13	2.4 ± 0.8	2.3 ± 0.7	2.8 ± 0.9	3.0 ± 1.5	2.3 ± 1.1	2.4 ± 1.0	2.8 ± 1.2	3.0 ± 1.1	3.1 ± 0.9	3.2 ± 0.7	3.5 ± 1.0	2.9 ± 1.2	3.8 ± 0.8^h^	3.6 ± 0.6^h^	3.6 ± 1.1^h^	2.9 ± 1.6^h^	11.6 ± 3.0	11.4 ± 2.4	12.7 ± 3.25	11.8 ± 3.8
S14	2.1 ± 0.8	2.4 ± 1.03	2.8 ± 1.1	2.6 ± 0.90	2.6 ± 1.2	2.6 ± 0.8	2.5 ± 0.9	3.2 ± 0.9	3.0 ± 01.1	3.1 ± 0.6	3.2 ± 0.5	3.1 ± 1.1	3.4 ± 0.7	3.2 ± 0.6	3.3 ± 0.6	2.7 ± 1.4	11.0 ± 3.1	11.4 ± 2.5	12.3 ± 2.5	11.7 ± 3.1
S15	2.6 ± 1.1	2.4 ± 0.8	2.79 ± 0.98	2.25 ± 0.97	2.3 ± 0.8	2.3 ± 0.6	2.7 ± 0.9	3.1 ± 0.9	2.9 ± 0.8	2.9 ± 0.8	3.2 ± 1.1	2.7 ± 1.1	3.2 ± 0.8	3.1 ± 0.9	3.2 ± 1.1	2.7 ± 1.1	11.1 ± 3.1	10.6 ± 2.8	11.7 ± 3.4	10.7 ± 3.0
S16	2.1 ± 0.9	2.5 ± 0.6	3.3 ± 1.1	2.6 ± 1.2	2.4 ± 1.3	1.88 ± 0.81	3.3 ± 1.3	3.0 ± 0.7	2.8 ± 1.1	2.8 ± 0.7	3.0 ± 1.11	3.2 ± 1.1	3.2 ± 1.1	2.8 ± 0.8	3.6 ± 1.3	2.9 ± 0.9	10.5 ± 3.4	8.8 ± 1.9	12.2 ± 4.3	11.7 ± 3.1
S17	2.4 ± 0.6	2.4 ± 0.7	2.9 ± 0.8	2.7 ± 1.1	2.4 ± 0.7	2.2 ± 0.7	3.2 ± 1.2	2.4 ± 0.9	2.8 ± 0.7	2.7 ± 0.6	3.3 ± 1.2	2.7 ± 1.1	3.2 ± 0.6	3.0 ± 0.9	3.4 ± 1.2	3.2 ± 1.1	10.8 ± 2.1	10.2 ± 2.3	12.8 ± 3.6	11.1 ± 2.3
S18	1.4 ± 0.6	2.3 ± 0.7	2.6 ± 0.5	2.6 ± 1.0	2.2 ± 0.7	2.4 ± 0.7	3.0 ± 1.0	2.5 ± 0.8	2.8 ± 0.7	2.6 ± 0.7	2.8 ± 1.1	2.6 ± 1.0	3.3 ± 0.6^h^	3.1 ± 0.7^h^	4.0 ± 2.8^h^	3.5 ± 0.7^h^	10.7 ± 1.4	10.4 ± 1.9	12.4 ± 3.6	11.2 ± 1.8
S19	2.4 ± 1.1	2.2 ± 0.6	2.5 ± 1.1	2.3 ± 0.8	2.1 ± 0.3	2.2 ± 0.4	2.7 ± 1.1	2.7 ± 1.0	2.6 ± 0.6	2.5 ± 0.8	2.7 ± 1.1	2.7 ± 1.0	3.2 ± 0.7	2.9 ± 0.6	2.9 ± 1.1	2.5 ± 0.7	10.2 ± 2.0	9.8 ± 1.9	10.8 ± 3.7	10.2 ± 2.0
S20	2.4 ± 0.6	2.4 ± 0.6	2.6 ± 0.7	2.4 ± 0.8	2.3 ± 0.7	2.2 ± 0.7	2.5 ± 0.8	2.5 ± 0.8	2.5 ± 0.8	2.6 ± 0.7	2.8 ± 0.9	2.4 ± 0.8	2.5 ± 0.8	2.8 ± 0.8	2.8 ± 0.8	2.2 ± 0.7	9.6 ± 2.8	10.0 ± 2.4	10.8 ± 2.7	9.6 ± 3.0
S21	2.5 ± 0.6	2.4 ± 0.6	2.6 ± 0.7	2.3 ± 0.6	2.2 ± 0.6	2.4 ± 0.6	2.5 ± 0.7	2.4 ± 0.7	2.6 ± 0.6	2.7 ± 0.6	2.9 ± 0.8	2.5 ± 0.7	2.8 ± 0.7	2.7 ± 0.7	2.7 ± 0.9	1.9 ± 0.8	10.1 ± 1.8	10.25 ± 1.77	10.6 ± 2.7	8.9 ± 2.3
S22	2.4 ± 0.8	2.6 ± 0.6	2.4 ± 0.8	2.3 ± 0.9	2.2 ± 0.6	2.3 ± 0.5	2.6 ± 0.8	2.2 ± 0.7	2.7 ± 0.6	2.7 ± 0.6	2.8 ± 1.0	2.2 ± 0.9	2.7 ± 0.9	2.7 ± 0.8	2.8 ± 1.1	1.9 ± 0.8	10.0 ± 2.0	10.4 ± 1.7	10.7 ± 3.1	8.7 ± 2.7
Overall	2.3 ± 0.3	2.5 ± 0.2	2.6 ± 0.3^∗^	2.4 ± 0.2	2.3 ± 0.3	2.3 ± 0.3	3.0 ± 0.4^¥^	2.8 ± 0.4^¥^	2.8 ± 0.3	2.8 ± 0.3	3.0 ± 0.3^b^	2.7 ± 0.3	3.2 ± 0.3^e^	3.1 ± 0.3^e^	3.2 ± 0.3^e^	2.7 ± 0.4	10.6 ± 0.8^i^	10.6 ± 0.9^i^	11.8 ± 1.0	10.6 ± 1.0^i^

*^∗^Higher values than RG and CG; ^¶^Higher values in 1st compared with 7th and 8th sessions; ^¥^Different from RuG and CG; *^$^*Different from 6th, 7th, 8th, 9th, 21st, and 22nd training sessions; ^§^Different from 2nd session; ^*a*^Different from 10th session; ^*b*^Different from RG and CG; ^*c*^Different from 3rd, 6th, 7th, 8th, 19th, 20th, and 22nd training sessions; ^*d*^Different from 13th and 14th training sessions; ^*e*^Different from CG; ^*f*^Different from 21st training session; ^*g*^Different from 21st To 22nd training sessions; ^*h*^Different from 20th, 21st, and 22nd sessions; ^*i*^Different from running group; ^*j*^Different from 7th, 8th, 19th, 20th, 21th, and 22nd training sessions; ^*k*^Different from 10th to 13th training sessions; a.u., arbitrary unit; S, training session; DOMS, delayed onset muscle soreness.*

Stress was different between groups (*F*_3,1254_ = 46.299 and *P* < 0.001). Lower values were observed for Randori (*P* < 0.001 for both comparisons) and Uchi-komi (*P* < 0.001 for both comparisons) compared with running and control groups. Moreover, a time-point effect was detected (*F*_21,1254_ = 3.005 and *P* < 0.001), with higher values for 1st compared with the 6th, 7th, 8th, 9th (*P* = 0.002, 0.002, 0.014, and 0.042, respectively), 21st (*P* = 0.028), and 22nd (*P* = 0.001) training sessions; higher values for 2nd compared with 7th training session (*P* = 0.036) and for 6th compared with 10th training session (*P* = 0.009).

Fatigue was different between groups (*F*_3,1254_ = 4.199 and *P* = 0.006). Higher values were observed for running than for Randori (*P* = 0.046) and control (*P* = 0.01) groups. Fatigue was also different across time-points (*F*_21,1254_ = 3.223 and *P* < 0.001), with higher values in 1st compared with 3rd (*P* = 0.003), 6th (*P* < 0.001), 7th (*P* < 0.001), 8th (*P* = 0.028), 19th (*P* = 0.012), 20th (*P* = 0.017), and 22nd training sessions (*P* = 0.002) and lower values in 6th compared with 13th (*P* = 0.004) and 14th training sessions (*P* = 0.027).

Delayed onset muscle soreness was different between groups (*F*_3,1254_ = 11.006 and *P* < 0.001). Higher values were observed for Randori, Uchi-komi, and running groups than for controls (*P* < 0.001, *P* = 0.001, and *P* < 0.001, respectively). A time-point effect was revealed (*F*_21,1254_ = 3.249 and *P* < 0.001), with lower values in 21st compared with 5th training session (*P* = 0.004), lower values in 21st and 22nd compared with 10th training session (*P* = 0.007 and *P* = 0.0013, respectively), lower values in 20th, 21st, and 22nd compared with 13th (*P* = 0.001, 0.001, and 0.001, respectively), and 18th training sessions (*P* = 0.009, 0.001, and 0.001, respectively).

Hooper index was different between groups (*F*_3,1254_ = 13.757 and *P* < 0.001). Higher values were observed for running compared with Randori (*P* = 0.002), Uchi-komi (*P* = 0.002) and control (*P* = 0.002) training groups. A time-point effect was detected (*F*_21,1254_ = 3.268 and *P* < 0.001), with higher values in 1st compared with 7th (*P* < 0.001), 8th (*P* < 0.001), 19th, 20th, 21th, and 22nd (*P* = 0.0027, 0.007, 0.002, and 0.002, respectively) training sessions, lower values for 7th in comparison with 10th and 13th (*P* = 0.0031 and 0.0038, respectively) training sessions.

Total quality recovery was different between groups (*F*_3,1254_ = 7.075 and *P* < 0.001, Table 3). Lower values were observed for running compared with Randori (*P* = 0.004), Uchi-komi (*P* = 0.001), and control groups (*P* = 0.002). A time-point effect was found (*F*_21,1254_ = 3.239 and *P* < 0.001), with higher values at last compared with 1st (*P* = 0.006), 3rd (*P* = 0.005), 5th (*P* = 0.045), 10th (*P* = 0.002), 13th (*P* = 0.028), and 17th (*P* = 0.004) training sessions. Additionally, the 8th session values were higher than those at 10th (*P* = 0.038), whereas 10th training session values were lower than 18th (*P* = 0.033) and 21st (*P* = 0.0038) training sessions.

**TABLE 3:** Physical enjoyment values [arbitrary unit] measured before and after intensified training and tapering periods (mean ± SD; RG *n* = 14; UG *n* = 16; RuG *n* = 17; and CG *n* = 12).

	Before intensified training	After intensified training	After tapering	Overall
Randori group	88 ± 6	88 ± 6	90 ± 7	89 ± 6
Uchi-komi group	88 ± 6	88 ± 7	89 ± 6	88 ± 6
Running group	80 ± 6	77 ± 7	78 ± 7	78 ± 7^∗^
Control group	86 ± 6	86 ± 6	86 ± 6	86 ± 6

*^∗^Different from other groups.*

Enjoyment was different between groups (*F*_3,171_ = 30.021 and *P* < 0.001). Lower values were observed for running compared with control (*P* < 0.001), Randori (*P* < 0.001), and Uchi-komi (*P* < 0.001) training groups. No significant time-point effect was observed for enjoyment.

## Discussion

The present study is the first to investigate the effects of intensified training followed by a tapering period using different training modalities on RPE, well-being, and physical enjoyment among young judo athletes. The main findings were that RPE was lower and well-being indices and recovery state were improved during the tapering period compared with the intense training period in all groups. Moreover, sleep quality, stress, fatigue, DOMS, and overall well-being (HI) was better in the Randori and Uchi-komi groups than in the running training group. Likewise, specific judo training modalities elicited higher values for TQR and physical enjoyment compared with running, whereas RPE did not differ across experimental groups.

Rating of perceived exertion represents a subjective tool used to inform and tailor workload during judo training (Magnani Branco et al., 2017). In the present study, RPE values decreased during the tapering period compared with the intense training period. This finding confirms that fatigue was reduced during the tapering phase, likely due to reduced training load. Moreover, subjective intensity measured by RPE was similar between running, Randori, and Uchi-komi groups, indicating a similar level of effort exerted regardless of training modality. Similar RPE between training modalities was previously observed in a study comparing Uchi-komi and upper- and lower-body cycle ergometer exercises (Magnani Branco et al., 2017). It is likely that the instruction to exert all-out effort, activation of large muscle groups, and the same time-structure for all training modalities contributed to similar subjective intensity among exercise modes.

Well-being indices (i.e., sleep, stress, fatigue, and DOMS) are used to monitor training and to avoid negative signs of intense training (Selmi et al., 2019). The results of present study indicated that sleep quality was worse at the beginning of training compared with the middle of the intense training period and was not different between training periods. Although HIIT volume was reduced during tapering, it appears that the changes in training load throughout the study did not substantially alter sleep quality of judo athletes. Similar results were recently observed by Lastella et al. (2018). In that study, sleep behavior was not affected by training load variation during a 14-day training camp in adolescent female elite basketball players. However, training modality may have a greater effect on sleep than training load. For example, Kölling et al. (2016) showed that HIIT resulted in sleep disturbances, whereas strength training group did not alter participants’ reported sleep quality. Similar outcomes were observed in the present study, where the poorest sleep quality was reported in the running group, and this was significantly lower than in the Randori and control groups. It is possible that non-sport-specific training impairs sleep quality to a greater degree than sport-specific training.

The results of present study showed that stress scores decreased across sessions, which may reflect a psychometric adaptation to training. Moreover, stress levels were lower during the tapering period. Similarly, Hooper et al. (1999) reported that the reduced training load during tapering reduced stress in athletes. Furthermore, lower stress scores were observed with Randori and Uchi-komi training in the present study, which suggests that the two types of judo-specific training modalities induced same amount of subjective stress, whereas intense non-sport-specific athletic training (running) resulted in increased psychological disturbance. Other studies examining psychometric responses to different training modalities in team sports athletes also found that intense general athletic exercise induced mood disturbances and stress (Saanijoki et al., 2015; Selmi et al., 2018b). The judo athletes in the present study were likely more motivated by combat opposition and technical creativity involved in sport-specific Randori and Uchi-komi training, possibly contributing to the low stress scores observed for these groups. When asked to attribute pleasure scores to exercises used in their training routine, Olympic judo athletes reported high values for judo-specific exercises, which were their main training activities (Franchini and Takito, 2014). Additionally, the results of the present study are consistent with those of Selmi et al. (2020) which reported that high-intensity training leads to positive affective responses when using sport-specific exercise modalities (i.e., small-sided games in soccer players) compared with a non-specific training modality. Therefore, it is likely that sport-specific exercises are preferred by and more motivating for athletes, resulting in lower stress scores compared with non-sport-specific exercises.

Fatigue decreased across training sessions, suggesting that athletes adapted to intensified training. Furthermore, fatigue recorded during the tapering period was lower than during intense training, indicating that reducing training volume successfully reduced fatigue. It was previously reported that decreasing training frequency may interact with other training variables such as volume and intensity (Bosquet et al., 2007). Likewise, the results of present study are consistent with findings of Papacosta et al. (2013) showing decreased fatigue and improved total mood scores during a 2-week tapering period in judo athletes. Reduced fatigue scores during tapering may reflect positive adaptive responses resulting from a physical performance enhancement as reported by Flatt et al. (2017). These authors reported that fatigue score returned to baseline values during a tapering period after intensified training leading to successful competition in swimmers. In the present study, fatigue was higher in the running versus the Randori and control groups, suggesting that running produced greater muscle fatigue. It is possible this increased fatigue was due to the novelty of running training since athletes were more familiar with other training modalities. Randori and Uchi-komi are sport-specific judo exercises performed for technical-tactical preparation as well as physical conditioning (Franchini and Takito, 2014).

Delayed onset muscle soreness scores were higher during the intensified training period and decreased during the tapering period, showing that muscle damage decreased with reduced training load (i.e., training volume). The results of the present study are in agreement with those of Papacosta et al. (2013) who found that muscle soreness was reduced by decreased training volume during tapering reflected by decreased in plasma creatine kinase (CK) activity (Papacosta et al., 2013). Furthermore, higher DOMS scores were recorded for all HIIT groups versus control in the present study. High-intensity or prolonged exercises lead to elevation of inflammatory responses, including during judo-specific training (Detanico et al., 2017). Moreover, judo is a dynamic sport that incorporates a variety of movements, such as eccentric muscle contractions and high impact forces, that can induce a high degree of muscle damage (Papacosta et al., 2013; Detanico et al., 2015). Thus, increase of muscle soreness in all HIIT groups may have resulted from high-intensity training sessions added to the regular judo training regimen. Franchini et al. (2016b) showed that muscle damage markers [i.e., CK, lactate dehydrogenase (LDH), aspartate aminotransferase (AST), and alanine aminotransferase (ALT)], assessed before and after four bouts of the Wingate test, increased only after upper- and lower-body high-intensity intermittent protocols were performed compared with pre-test values. Similarly, Franchini et al. (2016a) reported that CK, LDH, AST, and ALT increased after match simulations and that CK concentrations were the same for lower-body and Uchi-komi and decreased for upper-body (i.e., smaller quantity of active muscle mass) training group. In the present study, HI measured during tapering was lower than during the intense training period. HI is sensitive to variation in training load, where lower scores are associated with reduced workload (Fessi et al., 2016). The pattern of decrease in HI was similar to that of fatigue, suggesting that reduced fatigue due to decrease in volume and training frequency may have driven the reduction in HI during tapering. Results also indicated that HI for the running group was higher than that of the other groups. This finding suggests that increased HI during running training may be a sign of participants’ psychometric disruption (Selmi et al., 2018b). It has been reported that a poor (high) HI negatively influences athlete activity and produces emotional disturbances during training (Nedelec et al., 2014). Moreover, Hooper et al. (1995) indicated that HI can be used to detect excessive training stress. HI may also be used to avoid potential maladaptive physical and psychometric effects and is strongly associated with exercise performance and positive mood (Selmi et al., 2018b). Thus, higher DOMS and fatigue values recorded for running may drive higher HI ones compared with other training modalities.

Total quality of recovery is an easy and useful tool for monitoring recovery *status* of athletes (Kenttä and Hassmén, 1998). To the authors’ current knowledge, the impact of different training modalities, especially in combat sport athletes, on TQR has not yet been examined. The present study showed that TQR scores during the intense training period were lower than those measured during tapering, indicating that recovery *status* is positively related to training volume and frequency reduction. Similarly, Selmi et al. (2018b) reported that recovery state was strongly associated with reduced fatigue, low HI, and athletic training performance. Moreover, in the present study, TQR score was lower in running compared with other groups. One possible explanation is that quality of recovery is sensitive to well-being and emotional state of athletes as various emotional states can either interfere with or enhance the process of recovery (Sonnentag and Fritz, 2007; Nedelec et al., 2014). Furthermore, the repetition of high-intensity running with change of direction may have induced greater neuromuscular fatigue and impaired recovery to a greater degree than the sport-specific training modalities. The sport-specific modalities were based on maximizing technical capacities (i.e., Uchi-komi) or offensive and defensive skills (i.e., Randori), which appear to be more motivating. This may have positively affected the athletes’ emotional state, thereby enhancing their perceived recovery (Lundqvist and Kenttä, 2010).

Finally, selecting specific and motivating exercises is critically important for athletes, as physical enjoyment is strongly related to athlete motivation and is a key factor in commitment and performance (Selmi et al., 2018a). The results of the PACES questionnaire in the present study showed that judo athletes did not enjoy running as much as the other training modalities (i.e., Randori, Uchi-komi, and control groups). It is possible that running was perceived as less motivating and monotonous, which may have negatively affected mood and emotional state (Selmi et al., 2018a). For athletes, running training appears to be unpleasant, negatively perceived, and discouraging, which may explain lower physical enjoyment scores (Saanijoki et al., 2015). In contrast, specific judo training (i.e., Randori and Uchi-komi) elicited higher physical enjoyment. Previously, performing technical actions and specific combat skills produced better emotional responses compared with running training in Olympic judo athletes (Franchini and Takito, 2014). Furthermore, a recent study in soccer players demonstrated that exercise based on technical actions (e.g., small sided games) was perceived to be more enjoyable than general athletic training (Selmi et al., 2020). Altogether, physical enjoyment appears to reflect attitude, sport preference, and intrinsic motivation and varies according to exercise modality.

Although the present study has direct and considerable practical implications, some limitations should be highlighted. Firstly, participants’ maturation *status* was not controlled. In spite of being homogeneous in terms of chronological age, investigated 14–17 years old participants were still in the process of growth and maturation and that could have interfered with their perceived exertion, well-being, and recovery measures. Indeed, there were some height differences among groups in favor of experimental groups, in particular Randori and Uchi-komi, suggesting some growth differences. Future studies should pay more attention to participants homogeneity. Secondly, physical and physiological measures were not performed. Future studies should investigate changes in physical performance and physiological responses during intense training and tapering periods and their relationship with changes in psychometric state.

## Conclusion

The present study demonstrated that RPE was lower and well-being and recovery state improved during tapering following an intense training period. Moreover, running induced greater sleep disturbances, stress, fatigue, DOMS, and poorer well-being compared with sport-specific training groups. In contrast, Judo athletes enjoyed Randori and Uchi-komi more than running and perceived better recovery during sport-specific training. These differences cannot be explained by level of effort since RPE did not differ across groups. The results of present study suggest that coaches should monitor well-being indices, perceived exertion, and recovery state during different judo training periods. These variables are generally associated with performance of judo athletes. Moreover, to enhance motivation and engagement, it is recommended that coaches use specific judo exercises for athletes’ physical conditioning when practical. Finally, psychometric and recovery state adaptation to training occurred over 12 days of tapering and thus coaches should be aware that 12 days of tapering after 4 weeks of intensified training are effective during the competitive phase in judo.

## Data Availability Statement

The raw data supporting the conclusions of this article will be made available by the authors, without undue reservation.

## Ethics Statement

The studies involving human participants were reviewed and approved by the Comité de Protection des Personnes, Institut Supérieur des Sciences Infirmières de Sfax, Université de Sfax. Written informed consent to participate in this study was provided by the participants’ legal guardian/next of kin.

## Author Contributions

All authors listed have made a substantial, direct and intellectual contribution to the work, and approved it for publication.

## Conflict of Interest

The authors declare that the research was conducted in the absence of any commercial or financial relationships that could be construed as a potential conflict of interest. The reviewer DD declared a past co-authorship with one of the authors EF to the handling editor.

## Footnotes

1 A New View of Statistics: Effect Magnitudes, https://www.sportsci.org/resource/stats/effectmag.html

2 All 95% CI_*d*_s and *d*s are reported in Supplementary Table S1.

## References

[B1] Agostinho M. F. Moreira A. Julio U. F. Marcolino G. S. Antunes B. M. M. Lira F. S. (2017). Monitoring internal training load and salivary immune-endocrine responses during an annual judo training periodization. *J. Exerc. Rehabil.* 13 68–75. 10.12965/jer.1732850.425 28349036 PMC5332002

[B2] Agostinho M. F. Philippe A. G. Marcolino G. S. Pereira E. R. Busso T. Candau R. B. (2015). Perceived training intensity and performance changes quantification in judo. *J. Strength Cond. Res.* 29 1570–1577. 10.1519/JSC.0000000000000777 25436630

[B3] Bosquet L. Montpetit J. Arvisais D. Mujika I. (2007). Effects of tapering on performance: a meta-analysis. *Med. Sci. Sports Exerc.* 39 1358–1365. 10.1249/mss.0b013e31806010e0 17762369

[B4] Bromley S. Drew M. Talpey S. McIntosh A. Finch C. (2018). collecting health and exposure data in australian olympic combat sports: feasibility study utilizing an electronic system. *JMIR Hum. Factors.* 5:e27. 10.2196/humanfactors.9541 30305257 PMC6231822

[B5] Detanico D. Dal Pupo J. Franchini E. Dos Santos S. G. (2015). Effects of successive judo matches on fatigue and muscle damage markers. *J. Strength Cond. Res.* 29 1010–1016. 10.1519/JSC.0000000000000746 25426512

[B6] Detanico D. Dal Pupo J. Franchini E. Fukuda D. H. Dos Santos S. G. (2017). Effects of traditional judo training session on muscle damage symptoms. *J. Sports Med. Phys. Fitness* 57 872–878. 10.23736/S0022-4707.16.06320-9 27054353

[B7] Fessi M. S. Nouira S. Dellal A. Owen A. Elloumi M. Moalla W. (2016). Changes of the psychophysical state and feeling of wellness of professional soccer players during pre-season and in-season periods. *Res. Sports Med.* 24 375–386. 10.1080/15438627.2016.1222278 27574867

[B8] Flatt A. A. Hornikel B. Esco M. R. (2017). Heart rate variability and psychometric responses to overload and tapering in collegiate sprint-swimmers. *J. Sci. Med. Sport* 20 606–610. 10.1016/j.jsams.2016.10.017 27890479

[B9] Foster C. Florhaug J. A. Franklin J. Gottschall L. Hrovatin L. A. Parker S. (2001). A new approach to monitoring exercise training. *J. Strength Cond. Res.* 15 109–115. 10.1519/00124278-200102000-00019 11708692

[B10] Franchini E. Del Vecchio F. B. Matsushigue K. A. Artioli G. G. (2011). PhysiologicalProfiles of elite judo athletes. *Sports Med.* 41 147–166. 10.2165/11538580-000000000-00000 21244106

[B11] Franchini E. Julio U. Panissa V. Lira F. Agostinho M. Branco B. (2016a). Short-term low-volume high-intensity intermittent training improves judo-specific performance. *Arch. Budo* 12 219–229.

[B12] Franchini E. Julio U. F. Panissa V. L. G. Lira F. S. Gerosa-Neto J. Branco B. H. M. (2016b). High-intensity intermittent training positively affects aerobic and anaerobic performance in judo athletes independently of exercise mode. *Front. Physiol.* 7:268. 10.3389/fphys.2016.00268 27445856 PMC4923181

[B13] Franchini E. Takito M. Y. (2014). Olympic preparation in Brazilian judo athletes: description and perceived relevance of training practices. *J. Strength Cond. Res.* 28 1606–1612. 10.1519/JSC.0000000000000300 24149759

[B14] Freitas V. H. Nakamura F. Y. Miloski B. Samulski D. Bara-Filho M. G. (2014). Sensitivity of physiological and psychological markers to training load intensification in volleyball players. *J. Sports Sci. Med.* 13 571–579. 25177184 PMC4126294

[B15] Hooper S. L. Mackinnon L. T. (1995). Monitoring overtraining in athletes. recommendations. *Sports Med.* 20 321–327. 10.2165/00007256-199520050-00003 8571005

[B16] Hooper S. L. Mackinnon L. T. Howard A. (1999). Physiological and psychometric variables for monitoring recovery during tapering for major competition. *Med. Sci. Sports Exerc.* 31 1205–1210. 10.1097/00005768-199908000-00019 10449025

[B17] Hooper S. L. Mackinnon L. T. Howard A. Gordon R. D. Bachmann A. W. (1995). Markers for monitoring overtraining and recovery. *Med. Sci. Sports Exerc.* 27 106–112. 10.1249/00005768-199501000-00019 7898325

[B18] Julio U. F. Panissa V. L. G. Esteves J. V. Cury R. L. Agostinho M. F. Franchini E. (2017). Energy-system contributions to simulated judo matches. *Int. J. Sports Physiol. Perform.* 12 676–683. 10.1123/ijspp.2015-0750 27736247

[B19] Kendzierski D. DeCarlo K. J. (1991). Physical activity enjoyment scale: two validation studies. *J. Sport Exercise Psy.* 13 50–64. 10.1123/jsep.13.1.50

[B20] Kenttä G. Hassmén P. (1998). Overtraining and recovery. A conceptual model. *Sports Med.* 26 1–16. 10.2165/00007256-199826010-00001 9739537

[B21] Kölling S. Wiewelhove T. Raeder C. Endler S. Ferrauti A. Meyer T. (2016). Sleep monitoring of a six-day microcycle in strength and high-intensity training. *Eur. J. Sport Sci.* 16 507–515. 10.1080/17461391.2015.1041062 26062597

[B22] Lastella M. Vincent G. E. Duffield R. Roach G. D. Halson S. L. Heales L. J. (2018). Can sleep be used as an indicator of overreaching and overtraining in athletes? *Front. Physiol.* 29:436. 10.3389/fphys.2018.00436 29740346 PMC5928142

[B23] Lundqvist C. Kenttä G. (2010). Positive emotions are not simply the absence of the negative ones: development and validation of the emotional recovery questionnaire (EmRecQ). *Sport Psychol.* 24 468–488. 10.1123/tsp.24.4.468

[B24] Magnani Branco B. H. Lopes-Silva J. P. da Silva Santos J. Julio U. F. Panissa V. L. G. Franchini E. (2017). Monitoring training during four weeks of three different modes of high-intensity interval training in judo athletes. *Arch. Budo* 13 51–62.

[B25] Moalla W. Fessi M. S. Farhat F. Nouira S. Wong D. P. Dupont G. (2016). Relationship between daily training load and psychometric status of professional soccer players. *Res. Sports Med.* 24 387–394. 10.1080/15438627.2016.1239579 27712094

[B26] Nedelec M. McCall A. Carling C. Legall F. Berthoin S. Dupont G. (2014). The influence of soccer playing actions on the recovery kinetics after a soccer match. *J. Strength Cond. Res.* 28 1517–1523. 10.1519/JSC.0000000000000293 24172722

[B27] Osiecki R. Rubio T. B. G. Coelho R. L. Novack L. F. Conde J. H. S. Alves C. G. (2015). The total quality recovery Scale (TQR) as a proxy for determining athletes’ recovery state after a professional soccer match. *J. Exerc. Physiol. Online* 18 27–33.

[B28] Ouergui I. Kamzi S. Houcine N. Abedelmalek S. Bouassida A. Bouhlel E. (2019). Physiological responses during female judo combats: impact of combat area size and effort to pause ratio variations. *J. Strength Cond. Res.* 10.1519/JSC.0000000000003307 [Epub ahead of print]. 31343555

[B29] Papacosta E. Gleeson M. Nassis G. P. (2013). Salivary hormones, IgA, and performance during intense training and tapering in judo athletes. *J. Strength Cond. Res.* 27 2569–2580. 10.1519/JSC.0b013e31827fd85c 23249825

[B30] Saanijoki T. Nummenmaa L. Eskelinen J.-J. Savolainen A. M. Vahlberg T. Kalliokoski K. K. (2015). Affective responses to repeated sessions of high-intensity interval training. *Med. Sci. Sports Exerc.* 47 2604–2611. 10.1249/MSS.0000000000000721 26110694

[B31] Selmi O. Gonçalves B. Ouergui I. Levitt D. E. Sampaio J. Bouassida A. (2019). Influence of well-being indices and recovery state on the technical and physiological aspects of play during small-sided games. *J. Strength Cond. Res*. 10.1519/JSC.0000000000003228 [Epub ahead of print]. 31403575

[B32] Selmi O. Gonçalves B. Ouergui I. Sampaio J. Bouassida A. (2018a). Influence of well-being variables and recovery state in physical enjoyment of professional soccer players during small-sided games. *Res. Sports Med.* 26 199–210. 10.1080/15438627.2018.1431540 29376416

[B33] Selmi O. Marzouki H. Ouergui I. BenKhalifa W. Bouassida A. (2018b). Influence of intense training cycle and psychometric status on technical and physiological aspects performed during the small-sided games in soccer players. *Res. Sports Med*. 26 401–412. 10.1080/15438627.2018.1492398 29966440

[B34] Selmi O. Ouergui I. Levitt D. E. Nikolaidis P. T. Knechtle B. Bouassida A. (2020). Small-sided games are more enjoyable than high-intensity interval training of similar exercise intensity in soccer. *Open Access J. Sports Med.* 11 77–84. 10.2147/OAJSM.S244512 32210645 PMC7069497

[B35] Sonnentag S. Fritz C. (2007). The recovery experience questionnaire: development and validation of a measure for assessing recuperation and unwinding from work. *J. Occup. Health Psychol.* 12 204–221. 10.1037/1076-8998.12.3.204 17638488

[B36] Sterkowicz S. Zuchowicz A. Kubica R. (1999). Levels of anaerobic and aerobic capacity indices and results for the special fitness test in judo competitors. *J. Hum. Kinet.* 2 115–135.

[B37] Thum J. S. Parsons G. Whittle T. Astorino T. A. (2017). High-intensity interval training elicits higher enjoyment than moderate intensity continuous exercise. *PLoS One* 12:e0166299. 10.1371/journal.pone.0166299 28076352 PMC5226715

[B38] World Medical Association (2013). World medical association declaration of helsinki: ethical principles for medical research involving human subjects. *JAMA* 310 2191–2194. 10.1001/jama.2013.281053 24141714

